# *Rosmarinus officinalis* essential oil components promote anaesthesia in *Colossoma macropomum* (Cuvier, 1818) through behavioural and cardiorespiratory modulation

**DOI:** 10.1111/jfb.70648

**Published:** 2026-09-15

**Authors:** Lorena Cristina Nunes de Almeida, Axell Lins, Daniella Bastos de Araújo, Maria Klara Otake Hamoy, Marcelo Victor dos Santos Brito, Luana Vasconcelos de Souza, Sarah Farias Câmara, Luciana Eiro Quirino, Nilton Akio Muto, Moisés Hamoy

**Affiliations:** ^1^ Laboratory of Pharmacology and Toxicology of Natural Products ICB‐UFPA Belém Brazil; ^2^ Center for the Valorization of Bioactive Compounds from the Amazon ICB‐UFPA Belém Brazil

**Keywords:** cardiorespiratory physiology, *Colossoma macropomum*, electrocardiography, essential oils, fish anaesthesia, *Rosmarinus officinalis*

## Abstract

Anaesthetic management plays a crucial role in maintaining the welfare and physiological stability of fish during routine aquaculture and research procedures. The essential oil of *Rosmarinus officinalis* (ROEO) has emerged as a promising natural anaesthetic; however, its cardiophysiological effects in Amazonian species remain underexplored. This study assessed the behavioural, electrocardiographic and respiratory responses of *Colossoma macropomum* (tambaqui) exposed to ROEO, aiming to define safe and effective concentrations as a natural anaesthetic agent in a Neotropical teleost species. Juvenile tambaqui were exposed to immersion baths containing 90.60–181.20 mg L^−1^ ROEO for 5 min. Behavioural endpoints (latency to loss and recovery of postural reflex), electrocardiographic parameters (heart rate, QRS amplitude, R–R, P–Q and Q–T intervals) and opercular frequency were monitored. The oil composition was determined by gas chromatography. Statistical analyses were performed using one‐way ANOVA, followed by Tukey's test (*p* < 0.05). ROEO produced a clear dose‐dependent anaesthetic response, characterized by faster induction and prolonged recovery times at higher concentrations. Cardiac monitoring revealed reversible bradycardia (up to −56%), elongation of R–R and Q–T intervals and a slight reduction in QRS amplitude without arrhythmias. Opercular frequency decreased by up to 38% at 181.20 mg L^−1^, indicating moderate respiratory depression. All effects were reversible within the recovery period. Eucalyptol (47.5%), camphor (19.3%) and α‐pinene (12.2%) were the main phytoconstituents identified. *Rosmarinus officinalis* essential oil provides a safe, reversible and concentration‐dependent anaesthetic effect in *C. macropomum*, maintaining sinus rhythm and physiological recovery. These results highlight ROEO as a biodegradable, low‐toxicity and effective alternative to synthetic anaesthetics, reinforcing its applicability for sustainable and welfare‐oriented aquaculture practices.

## 1 INTRODUCTION

The use of anaesthesia in aquaculture and scientific research involving fish plays a fundamental role in maintaining the health and welfare of these animals. Handling procedures, both for productive and experimental purposes, often involve invasive techniques that can cause stress and physical harm to fish (Aktop et al., 2019; Barbas et al., 2021; Brønstad, 2022; Kheawfu et al., 2021; Reis et al., 2024). In this context, anaesthetic use is essential to minimize such impacts and ensure more humane practices, providing safety for both animals and operators or consumers, while reflecting a commitment to ethical principles in animal management (Aydın & Barbas, 2020; Mocho, 2024; Rairat et al., 2021; Schroeder et al., 2021).

Synthetic anaesthetics, such as MS‐222 (tricaine methanesulfonate) and Benzocaine, are widely used in aquaculture. However, their use has been associated with undesirable side effects, including respiratory and cardiac depression, as well as the potential accumulation of residues in fish tissues, compromising aquaculture product quality and causing adverse effects on animal health and the environment (Can & Sümer, 2019; Mirghaed et al., 2018; Neiffer, 2021). Consequently, there has been growing interest in safer and more sustainable alternatives, such as plant‐derived natural anaesthetics, which offer a more favourable profile in terms of toxicity, biodegradability and economic accessibility (Bianchini et al., 2017; de Araújo et al., 2021; Elmas & Karadal, 2022; Rucinque et al., 2021; Seyidoglu & Yagcilar, 2020).

Among the promising natural anaesthetics is the essential oil of *Rosmarinus officinalis*, a perennial plant from the Lamiaceae family widely recognized for its diverse pharmacological properties, including antibacterial, antidiabetic, anti‐inflammatory, antitumour and antioxidant actions (Al Zuhairi et al., 2020; Al‐Maharik et al., 2022; Andrade et al., 2018; Li Pomi et al., 2023; Sharma et al., 2024). Among its main components, 1,8‐cineole (eucalyptol) stands out in experimental studies due to its ability to act on the central nervous system, activating transient cation channels that promote desensitization and result in anaesthetic effects (Caceres et al., 2017; Cai et al., 2021; Dhouibi et al., 2023; Khumpirapang et al., 2018; Sadeh et al., 2019). In addition to its anaesthetic properties, 1,8‐cineole also influences autonomic cardiovascular regulation, demonstrating effects such as bradycardia and reduced blood pressure, underscoring its therapeutic potential (Lahlou et al., 2002; Moon et al., 2014).

In fish experiments, 1,8‐cineole has proven to be an effective anaesthetic, with induction and recovery times directly dependent on the concentration used. In common carp (*Cyprinus carpio*), results indicate that higher concentrations reduce induction time, highlighting the potential of 1,8‐cineole as a promising natural anaesthetic for use in aquaculture (Hoseini et al., 2020; Mazandarani & Hoseini, 2017; Mirghaed et al., 2018). Furthermore, studies in rats have shown that intravenous administration of 1,8‐cineole significantly reduces mean arterial pressure and heart rate in a dose‐dependent manner, also indicating its effect on the cardiovascular system (Lahlou et al., 2002). Despite these advances, there remains a research gap concerning the cardiovascular effects of 1,8‐cineole in different fish species, which justifies the need to expand its evaluation.

The present study aims to investigate the anaesthetic effect of *Rosmarinus officinalis* essential oil in *Colossoma macropomum* (tambaqui), one of the most widely farmed species in Brazilian aquaculture. The study sought to determine optimal dose–response relationships for different concentrations of the oil and to assess cardiovascular safety and behavioural effects in fish during the induction and recovery phases of anaesthesia. This study aims to provide a safer and more sustainable alternative to synthetic anaesthetics, contributing to animal welfare and promoting more ethical and sustainable practices in aquaculture.

## 2 MATERIALS AND METHODS

### 2.1 Experimental animals

The subjects used were *Colossoma macropomum* tambaqui (*n* = 171), housed in aquariums in the Experimental Animal Facility of the Laboratory of Pharmacology and Toxicology of Natural Products at the Federal University of Pará (ICB/UFPA). The environment was maintained at a controlled temperature (25–27°C) with a 12 h light: 12 h dark photoperiod. The fish were fed commercial feed (32% protein) twice daily until satiation. Simultaneously, uneaten food and faeces were removed by siphoning, and the water was partially renewed (approximately 40% of tank volume) with water from the same source. During the acclimatization period (20 days), water quality variables such as water temperature (°C) (26.5°C) and pH (pH = 7.5) were monitored. The project was previously approved by CEUA‐UFPA under registration 5,846,030,624 (ID 002640).

### 2.2 Organoleptic properties and chromatographic analysis

Rosemary essential oil (*Rosmarinus officinalis*)—ROEO—was acquired from Harmonie Aromatherapy (Florianópolis, SC, Brazil, CNPJ: 11.938.821/0001‐90). The oil was extracted by steam distillation, and analysis was conducted by gas chromatography using an AGILENT 7820A Gas Chromatograph under the following conditions: Column: RXi‐5MS 30 m × 0.25 mm × 0.25 μm (Restek). Temperature program: Column at 50°C (0 min), increasing at 3°C/min to 200°C. Injector: 200°C, split ratio: 1/50. FID detector: 220°C. Injection volume: 1 μL (1% in ethyl acetate). The density of *Rosmarinus officinalis* essential oil was measured at 0.906 g/cm^3^. The phytoconstituents of the oil were identified as follows: Eucalyptol (47.5%), Camphor (19.3%), α‐pinene (12.2%), β‐pinene (7.8%) and β‐caryophyllene (4.6%) (Figures 1 and 2, Table 1).

**FIGURE 1:**
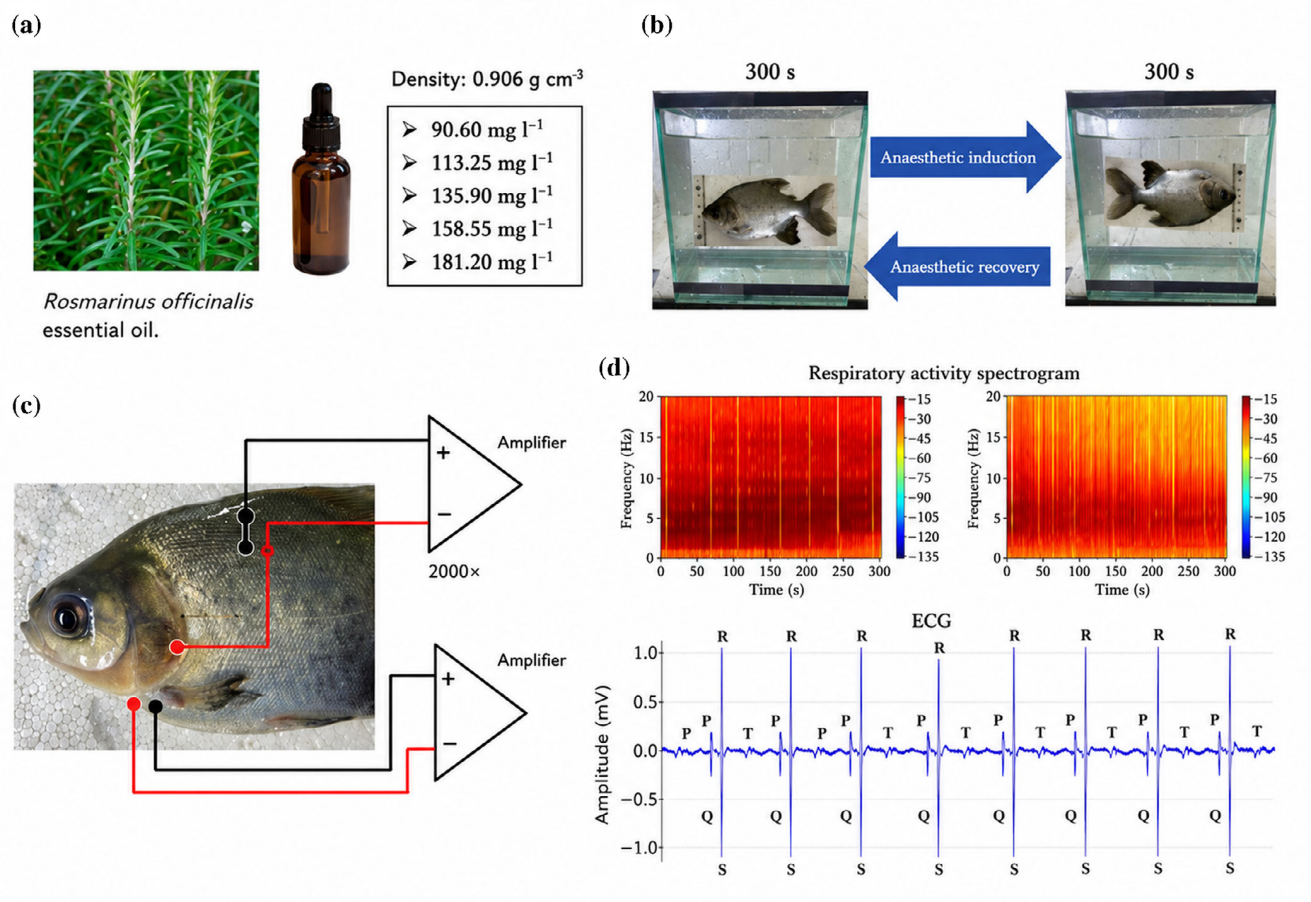
Experimental design, immersion bath in *Rosmarinus officinalis* essential oil with a density of 0.906 g/cm^3^, reduced to immersion bath at 90.60, 113.25, 135.90, 158.55, 181.20 (a); Immersion bath in juvenile *Colossoma macropomum* for anaesthetic induction and recovery (b); Electrode placement for acquisition of opercular beat signal and electrocardiogram (c); Spectrographic recordings of the opercular beat demonstrating anaesthetic induction and electrocardiographic recording demonstrating the graph elements of the ECG (d).

**FIGURE 2:**
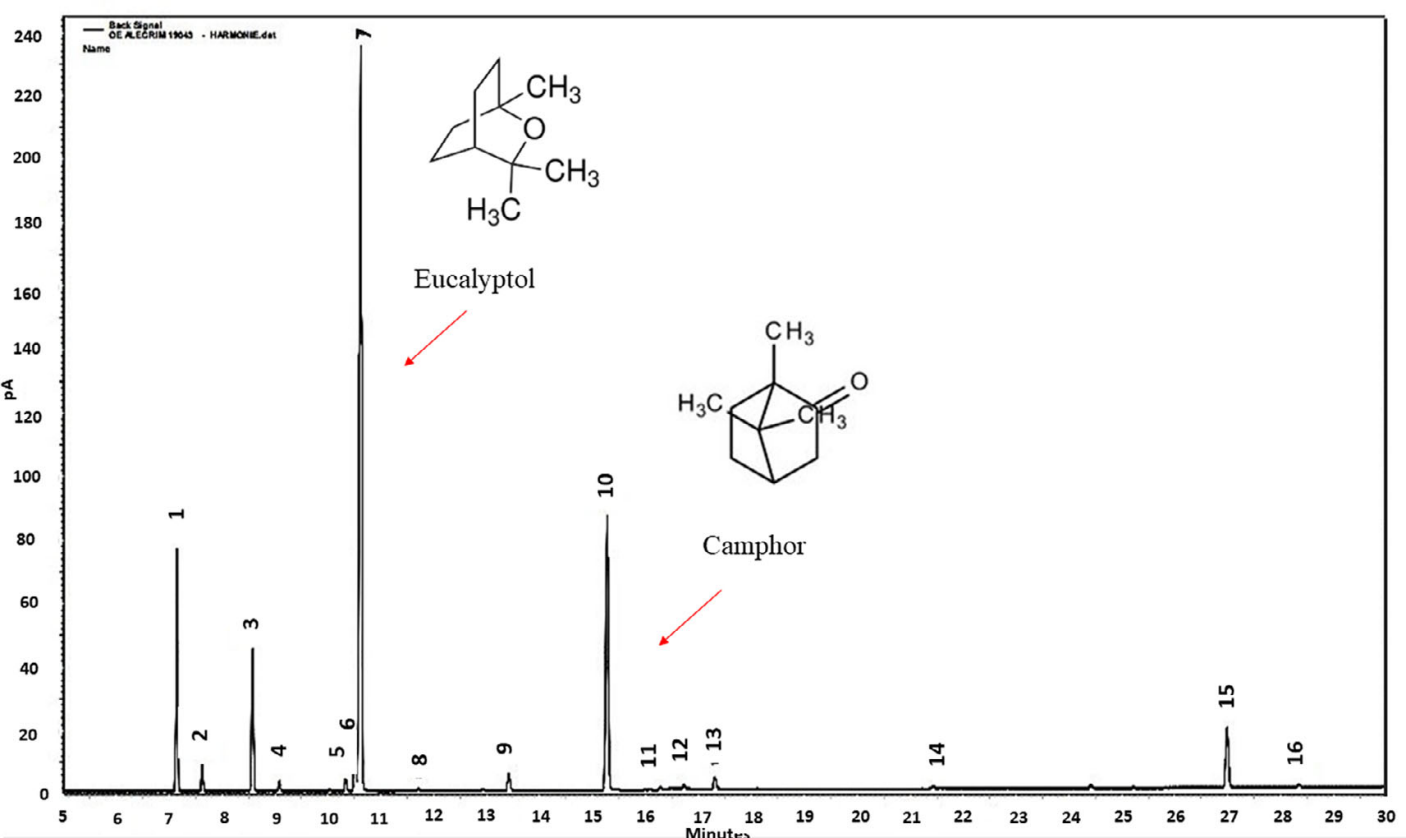
Chromatogram of the *Rosmarinus officinalis* essential oil sample from the Chromatography Laboratory, Department of Chemistry – Federal University of Minas Gerais, Belo Horizonte, Brazil.

**TABLE 1:** Chemical composition of *Rosmarinus officinalis* essential oil.

Retention index	Identification	Percentage (%)	Peak
913	Alpha‐pinene	12.2	1
925	Camphene	1.4	2
950	Beta‐pinene	7.8	3
963	Myrcene	0.5	4
995	Para‐cymene	0.7	5
1000	Limonene	2.3	6
1003	Eucalyptol	47.5	7
1031	Gamma terpinene	0.1	8
1073	Linalool	1.2	9
1121	Camphor	19.3	10
1148	Borneol	0.1	11
1159	Terpinen‐4‐ol	0.3	12
1174	Alpha terpineol	1.0	13
1281	Bornyl acetate	0.2	14
1425	Beta‐caryophyllene	4.6	15
1461	Humolene	0.2	16
	Others	0.2	

### 2.3 Experimental design

#### 2.3.1 Experiment with Rosmarinus officinalis essential oil (ROEO)

Juvenile *tambaqui* (26.18 ± 3.5 g) were randomly assigned to the following treatments: (a) Control; (b) vehicle group (fish subjected to immersion bath with 2 mL of 70% alcohol diluted in 1 L of aquarium water); (c) fish treated with ROEO 90.60 mg L^−1^; (d) 113.25 mg L^−1^; (e) 135.90 mg L^−1^; (f) 158.55 mg L^−1^ and (g) 181.20 mg L^−1^. All fish underwent anaesthetic induction with a contact period of 5 min, followed by observation during recovery for 5 min in water without ROEO. For each recording, *n* = 9 per treatment was used (immersion bath with ROEO and recovery post‐immersion bath), totaling 171 animals (Figure 1a,b).

#### 2.3.2 Experiment 1—Behavioural analysis of anaesthetic induction and recovery

Considering the exposure time for the following ROEO treatments: (a) 90.60 mg L^−1^, (b) 113.25 mg L^−1^, (c) 135.90 mg L^−1^, (d) 158.55 mg L^−1^ and (e) 181.20 mg L^−1^ (*n* = 9 per treatment), the latency to the behaviour of postural reflex loss (characterized by lateral recumbency) was evaluated. Following removal from ROEO contact, the latency for recovery of postural reflex was recorded (Figure 1b).

#### 2.3.3 Experiment 2—Electrocardiogram (ECG) analysis

For cardiac function analysis and monitoring, groups were divided as follows: (a) Control group; (b) vehicle group; (c) treated group with ROEO 90.60 mg L^−1^; (d) 113.25 mg L^−1^; (e) 135.90 mg L^−1^; (f) 158.55 mg L^−1^ and (g) 181.20 mg L^−1^ (a total of 63 animals). Electrodes were made from 0.3 mm diameter and 10 mm long 925 silver electrodes and subsequently isolated with liquid insulation. Electrodes were non‐conjugated. The reference electrode positioning followed the cardiac vector indication (negative pole at the cardiac base and positive pole at the apex). The reference electrode was fixed to the ventral portion of the left opercular opening 0.2 mm past the opercular cavity. The recording electrode was inserted 2.0 mm from the right opercular opening, capturing the signal on lead D1. The electrodes were connected to a high‐impedance amplifier (Grass Technologies, Model P511) for ECG recording, enabling analysis of heart rate (bpm), QRS complex amplitude (mV), QRS complex duration (ms) and R‐R (ms), P‐Q (ms) and Q‐T intervals (ms) (Figure 1c,d). For electrode implantation, the animals were gently removed from the aquaria and wrapped in a damp sponge; subsequently, the opercular opening was exposed, and a cotton swab soaked in 2% lidocaine was applied to the electrode insertion site. Only after this procedure were the electrodes inserted to acquire the cardiac signal. The volume of previously oxygenated aquarium water was 2 L for both induction and recovery, and it was renewed for each fish undergoing treatment.

#### 2.3.4 Experiment 3—Opercular activity recording

For opercular activity analysis, 925 silver electrodes were made with a 0.5 mm diameter and 15 mm length, conjugated at a 7 mm distance and isolated with liquid insulation. The electrodes were placed at the centre of the right opercular opening to record opercular beating, assessing the frequency (beats per minute) and power of opercular beating (mV^2^/Hz) (a total of 63 animals) (Figure 1c,d).

### 2.4 Recording and analysis of data

The electrodes were connected to a digital data acquisition system via a differential high‐impedance amplifier (Grass Technologies, Model P511), set with 0.3 to 300 Hz filtering, 2000× amplification and monitored by an oscilloscope (ProteK, Model 6510). Recordings were continuously digitized at a 1 KHz rate on a computer equipped with a data acquisition board (National Instruments, Austin, TX) and stored on a hard drive for further processing via specialized software (LabVIEW express). The acquired signals were analysed using a tool developed in Python version 2.7. Numpy and Scipy libraries were used for mathematical processing, while Matplotlib was used for graph plotting. The graphical interface was created with the PyQt4 library.

### 2.5 Statistical analysis

After confirming normality and homogeneity of variances with the Kolmogorov–Smirnov and Levene tests, respectively. The Kolmogorov–Smirnov (KS) test was performed in Python using the SciPy library. Comparisons of mean power values were made via one‐way ANOVA, followed by Tukey's test. GraphPad Prism® 8 software was used for the analyses, with *p* < 0.05, *p < 0.01 and **p < 0.001 considered statistically significant in all cases.

## 3 RESULTS

The behavioural analysis showed that ROEO caused a concentration‐dependent loss of postural reflex, with higher doses resulting in shorter latency for reflex loss onset. Fish exposed to 90.60 mg L^−1^ exhibited an average postural reflex loss at 214.1 ± 16.66 s, which was similar to the group treated with 113.25 mg L^−1^ (*p* = 0.060). However, these were longer than in the other groups. The group treated with 135.90 mg L^−1^ (151.4 ± 18.76 s), 158.55 mg L^−1^ (131.2 ± 8.89 s) and 181.20 mg L^−1^ (110.9 ± 13.57 s) were significantly different (Figure 3a).

**FIGURE 3:**
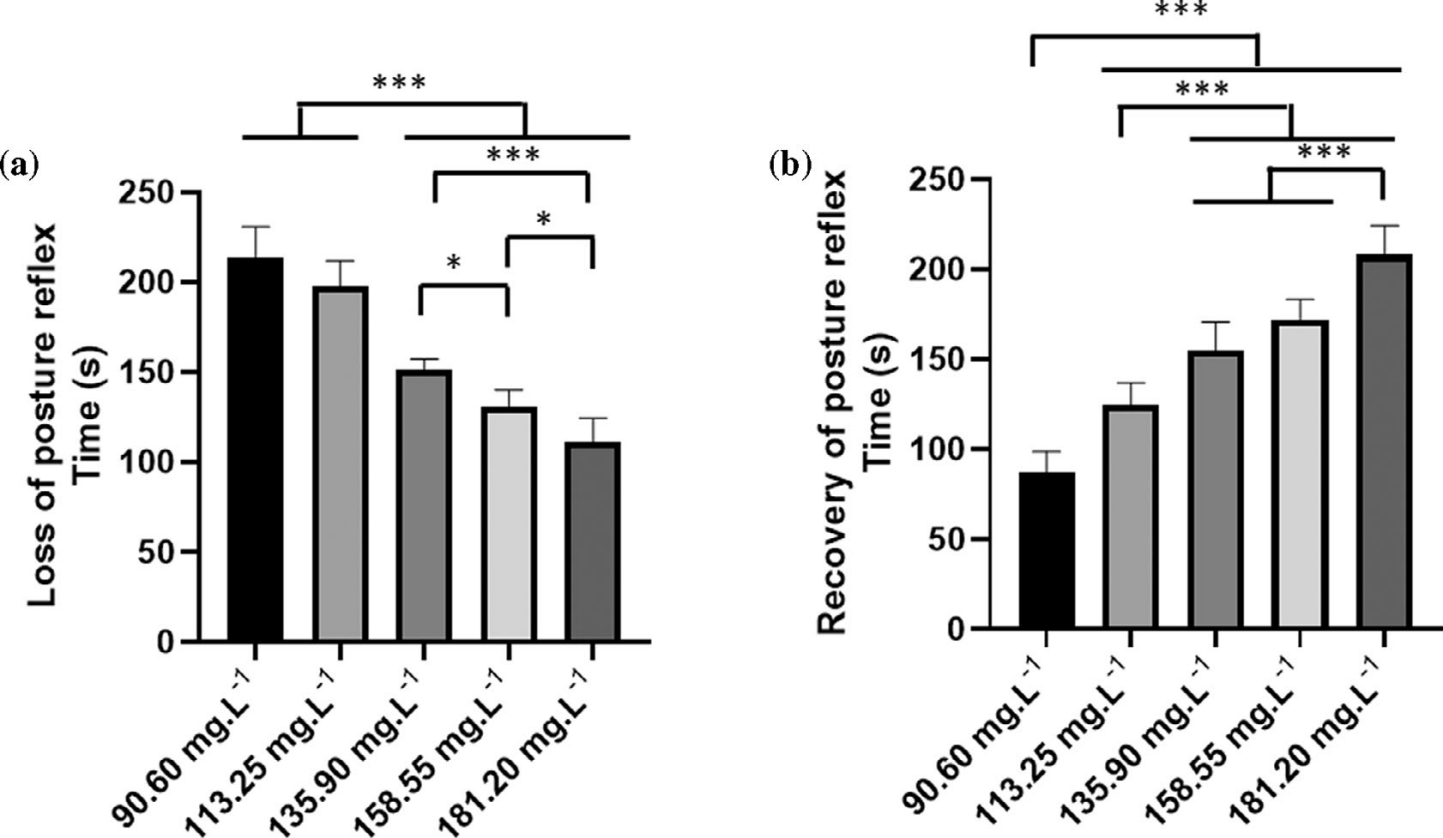
Average latencies (s) for loss of postural reflex during immersion baths with different ROEO treatments (a). Recovery of postural reflex after contact with different concentrations of ROEO (b). (ANOVA followed by Tukey's test; *p < 0.05, **p < 0.01 and ***p < 0.001).

The recovery of postural reflex in the group treated with 90.60 mg L^−1^ ROEO occurred in 87.0 ± 11.75 s and was shorter than in the other groups: 113.25 mg L^−1^ (124.6 ± 12.38 s), 135.90 mg L^−1^ (154.7 ± 15.80 s), 158.55 mg L^−1^ (172.2 ± 11.01 s) and 181.20 mg L^−1^ (208.1 ± 16.13 s). All groups showed recovery time dependent on the concentration used, with higher concentrations resulting in longer recovery times, indicating a slower reversibility of the effect for groups that received higher concentrations (Figure 3b).

The normal electrocardiogram of the tambaqui exhibited a sinus rhythm with an average frequency of 86.89 ± 2.47 bpm. The P wave, QRS complex and T wave can be identified (Figure 4a–c). The intervals between R‐R deflections, as well as the P‐Q and Q‐T intervals, remained regular considering the normal cardiac parameters. Thus, the interference of increasing doses of ROEO was measured. For the control group, the heart rhythm was sinusoidal during the 5‐min recording (Figure 4).

**FIGURE 4:**
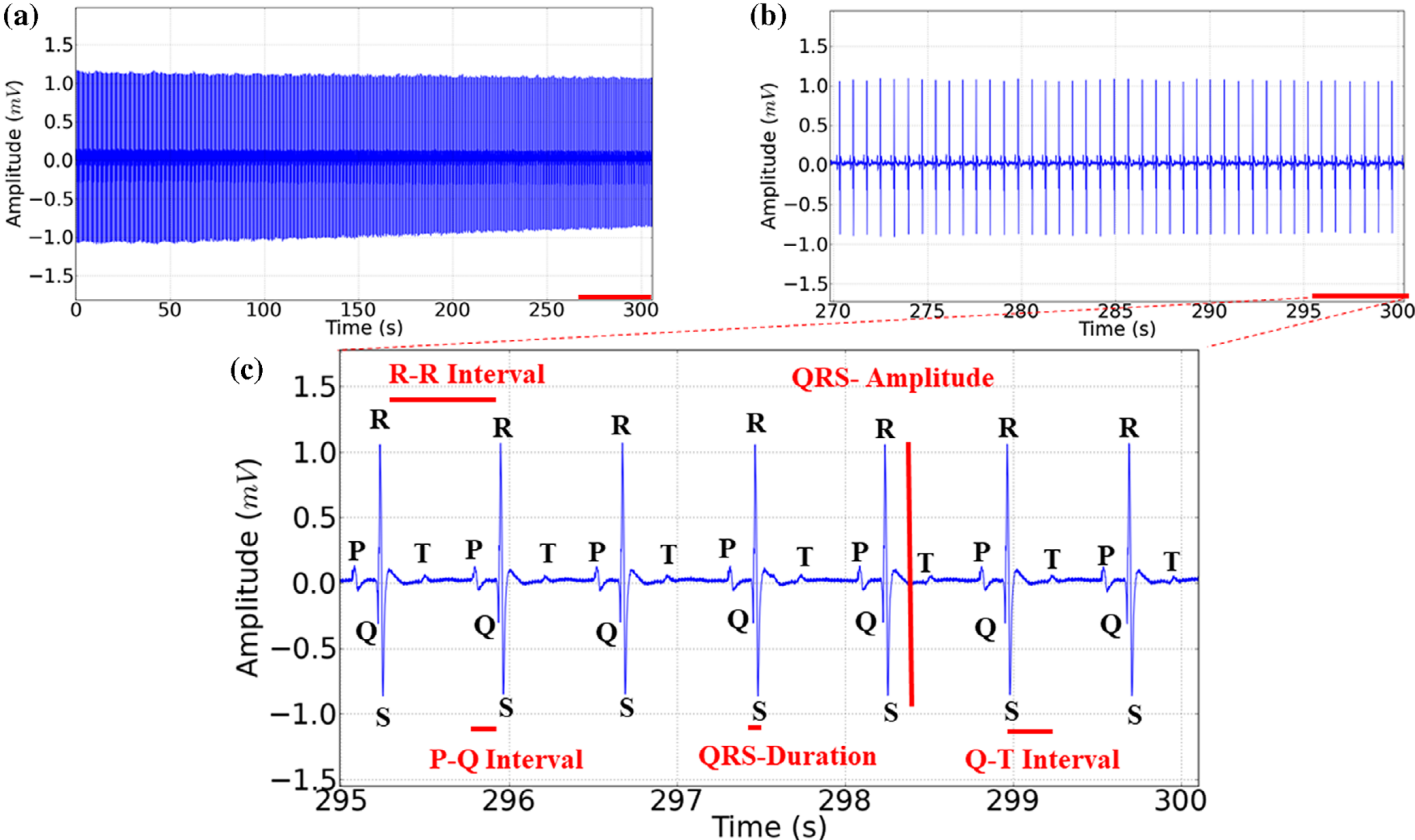
Normal electrocardiographic recording (ECG) of the tambaqui, *Colossoma macropomum* (a); enlarged electrocardiogram during the final 30 s of recording (270–300 s) (b); amplification of the last 5 s of the recording, demonstrating the morphological elements: P wave, QRS complex and T wave, as well as analysed elements: Heart rate (bpm), P‐Q interval, R‐R interval (s), duration of QRS (s), Q‐T interval (s) (c).

The cardiac activity in the control group showed a mean frequency of 86.89 ± 2.47 bpm, which was similar to the vehicle group (*p* = 0.999), with a sinus rhythm and all cardiac deflections present on the electrocardiogram (Figure 5a). In the 30‐s amplification, all graph elements were visible: P wave, QRS complex and T wave in a 5‐s amplification, allowing for the evaluation of intervals during the immersion bath in ROEO and their recovery. This enabled the identification of atrial activity represented by P waves, ventricular activity by QRS complexes and ventricular repolarization represented by T waves (Figure 5a–f).

**FIGURE 5:**
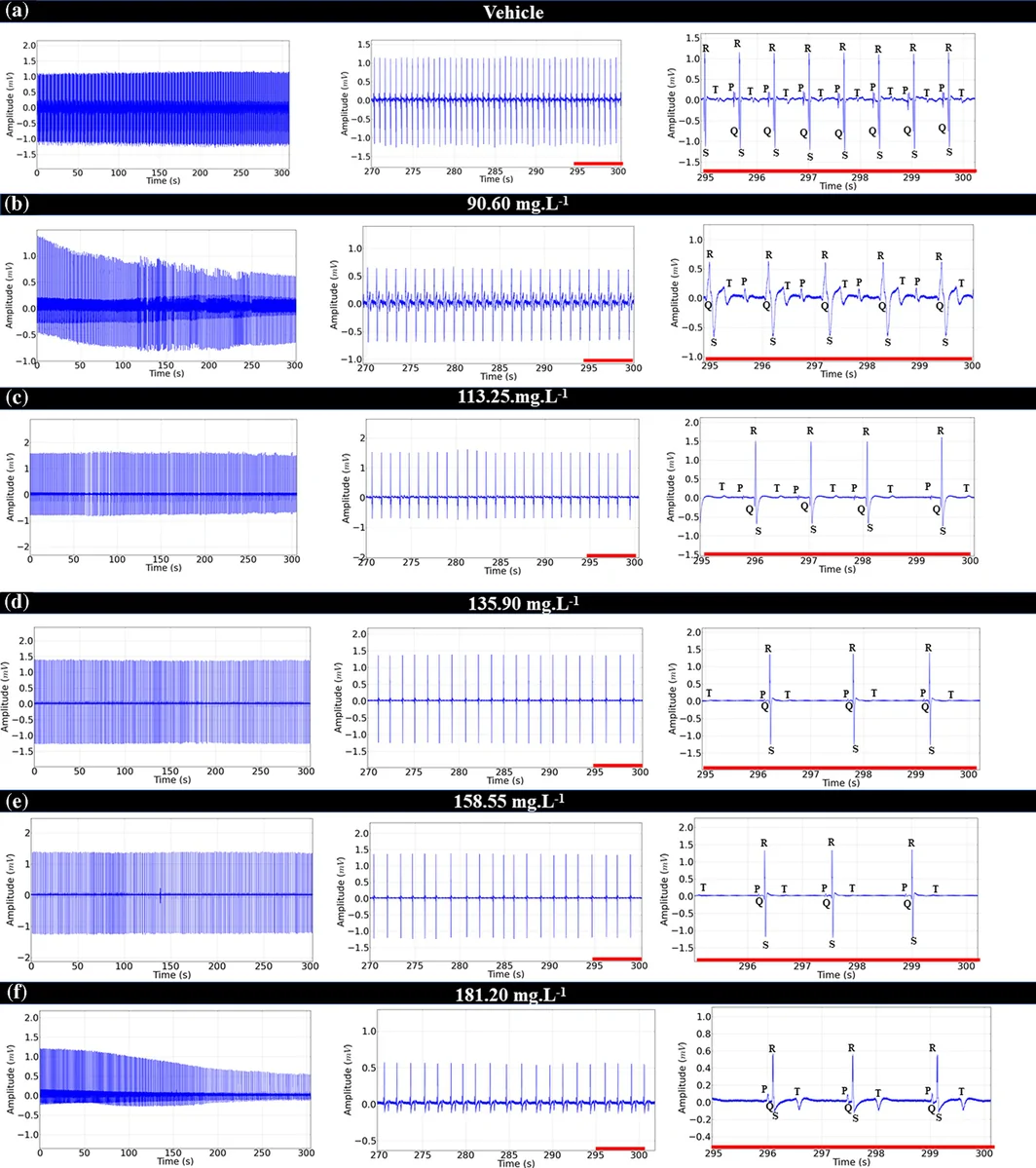
Electrocardiographic recordings demonstrating cardiac activity in juvenile *Colossoma macropomum* (left), amplification of the last 30 s of the 5‐min recording (270–300 s) (centre), showing the evaluated graph elements, including the P wave, QRS complex and T wave (amplification of 5 s) (right). Cardiac activity in juvenile *Colossoma macropomum* during immersion with the vehicle, with a description of the cardiac graph elements (a); groups treated with immersion at different concentrations of ROEO, along with their amplification and identification of cardiac deflections: 90.60 mg L^−1^ (b), 113.25 mg L^−1^ (c), 135 mg L^−1^ (d), 158.55 mg L^−1^ (e) and 181.20 mg L^−1^ (f).

During immersion treatment with 90.60 mg L^−1^, the fish showed a 40.66% decrease in heart rate compared to the control group (Figure 5b). The group treated with 113.25 mg L^−1^ of ROEO exhibited a 45.78% decrease (Figure 5c), while the group treated with 135.90 mg L^−1^ showed a 55.49% reduction (Figure 5d). The 157.55 mg L^−1^ group decreased by 56.51% (Figure 5e), and the group treated with 181.20 mg L^−1^ exhibited a 56.77% reduction (Figure 5f). The treated fish presented bradycardia while maintaining sinus rhythm (Figure 5b–f).

The heart rate was significantly affected by the increasing concentrations of ROEO. The control group had an average heart rate of 86.89 ± 2.47 bpm, and the vehicle group showed 86.67 ± 3.46 bpm, both being similar (*p* = 0.999). However, these values were higher than those in the treated groups. The group treated with 90.60 mg L^−1^ had an average heart rate of 51.56 ± 2.60 bpm, which was higher than the other treated groups. The group treated with 113.25 mg L^−1^ had an average of 47.11 ± 1.76 bpm, which was also higher than the groups treated with the higher doses. The group treated with 135.90 mg L^−1^ had an average of 38.67 ± 2.44 bpm, which was like the groups treated with 158.55 mg L^−1^ and 181.20 mg L^−1^ (*p* = 0.966) (Figure 6a).

**FIGURE 6:**
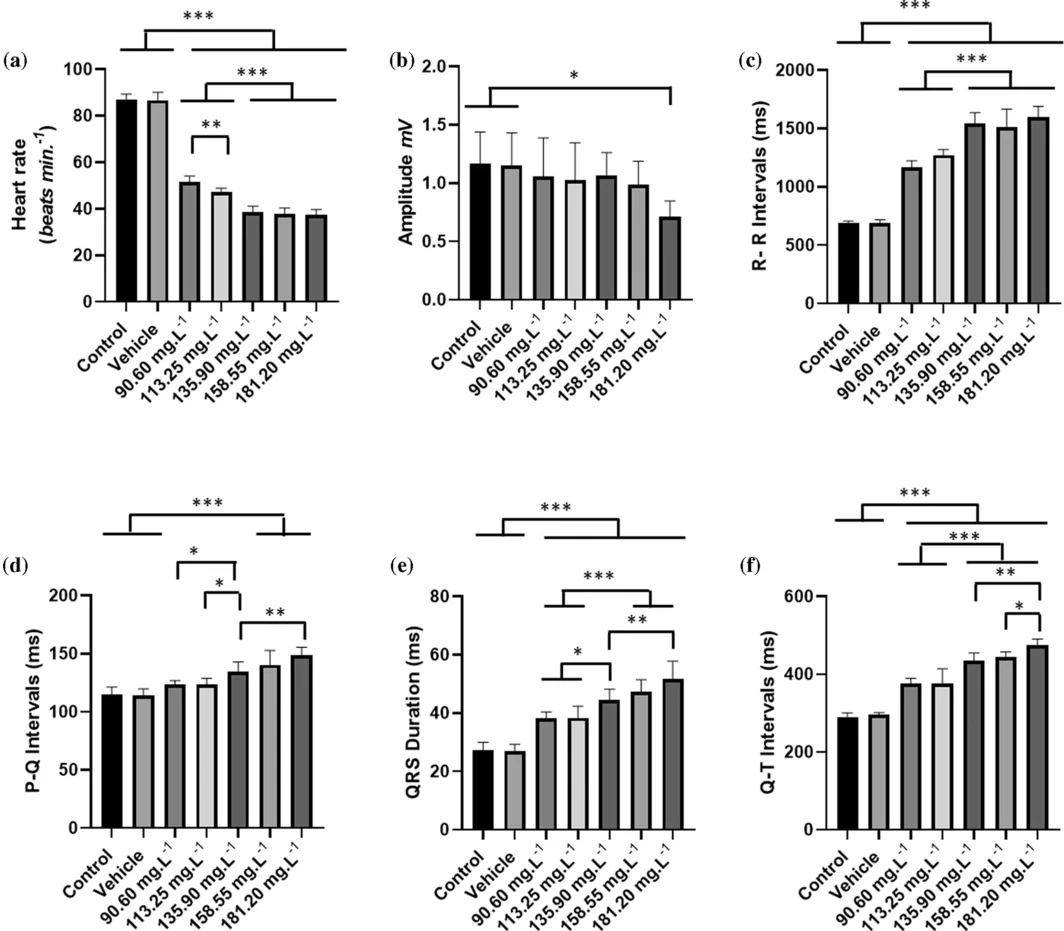
Average values of heart rate in beats per minute (bpm) (a), average amplitude values of the QRS complex (mV) (b), average RR interval values (ms) (c), average PQ interval (ms) (d), duration of the QRS complex (ms) (e) and QT interval (ms) (f) during exposure to ROEO at concentrations of 90.60 mg L^−1^, 113.25 mg L^−1^, 135.90 mg L^−1^, 158.55 mg L^−1^ and 181.20 mg L^−1^ (ANOVA followed by Tukey's test; *p < 0.05, **p < 0.01, ***p < 0.001; *n* = 9).

The average amplitude of the QRS complex for the control group was 1.168 ± 0.27 mV, and for the vehicle group, it was 1.15 ± 0.27 mV, which were similar to the groups treated with 90.60 mg L^−1^, 113.25 mg L^−1^ and 135.90 mg L^−1^, as well as 158.55 mg L^−1^ (*p* = 0.747). The control and vehicle groups had greater amplitudes than the group treated with 181.20 mg L^−1^, which measured 0.713 ± 0.134 mV (Figure 6b).

The average RR interval for the control group was 696.7 ± 19.33 ms, which was similar to the vehicle group (*p* = 0.999) and was shorter compared to the other treated groups. The group treated with 90.60 mg L^−1^ had an average RR interval of 1166 ± 58.31 ms, like the group treated with 113.25 mg L^−1^ (*p* = 0.1106) and was shorter than the other treated groups. The group treated with 135.90 mg L^−1^ had an RR interval of 1545 ± 91.82 ms, which was similar to the groups treated with 158.55 mg L^−1^ and 181.20 mg L^−1^ (*p* = 0.2725) (Figure 6c).

The average PQ interval for the control group was 115.1 ± 6.27 ms and showed no difference from the vehicle group (*p* = 0.999), as well as from the groups treated with 90.60 mg L^−1^ and 113.25 mg L^−1^ (*p* = 0.1060). The group treated with 135.90 mg L^−1^ had an average of 134.8 ± 8.21 ms, and the group treated with 158.55 mg L^−1^ was similar (*p* = 0.6530). The group treated with 158.55 mg L^−1^ was also like the group treated with 181.20 mg L^−1^ (*p* = 0.2505) (Figure 6d).

The average duration of the QRS complex for the control group during induction was 27.22 ± 2.72 ms, which was similar to the vehicle group (*p* = 0.999) but shorter than the other groups. The groups treated with 90.60 mg L^−1^ (38.11 ± 4.06 ms) were like the group treated with 113.25 mg L^−1^ (*p* = 0.9999). The group treated with 135.90 mg L^−1^ (44.33 ± 3.87 ms) was like the group treated with 158.55 mg L^−1^ (*p* = 0.6513). The group treated with 181.20 mg L^−1^ (51.67 ± 6.083 ms) was greater than the other groups but was like the group treated with 181.20 mg L^−1^ (*p* = 0.2251) (Figure 6e).

For the control group, the average QT interval during induction was 290.3 ± 10.51 ms, which was similar to the vehicle group (*p* = 0.9967) and shorter than the other groups. The group treated with 90.60 mg L^−1^ had an average QT interval of 375.7 ± 13.59 ms, and the 113.25 mg L^−1^ group was not significantly different (*p* = 0.999). The group treated with 135 mg L^−1^ (435.7 ± 18.91 ms) was like the group treated with 158 mg L^−1^ (*p* = 0.9525). The group treated with 181.20 mg L^−1^ (474.1 ± 16.44 ms) was greater than the other groups (Figure 6f).

During the recovery period following exposure to the ROEO concentrations of 90.60 mg L^−1^, 113.25 mg L^−1^, 135.90 mg L^−1^, 158.55 mg L^−1^ and 181.20 mg L^−1^, a reversibility of the electrocardiographic alterations was observed (Figure 7a–e), occurring more slowly in the groups treated with higher concentrations. The cardiac rhythm remained sinusoidal, with a gradual increase in heart rate approaching levels observed in the control group.

**FIGURE 7:**
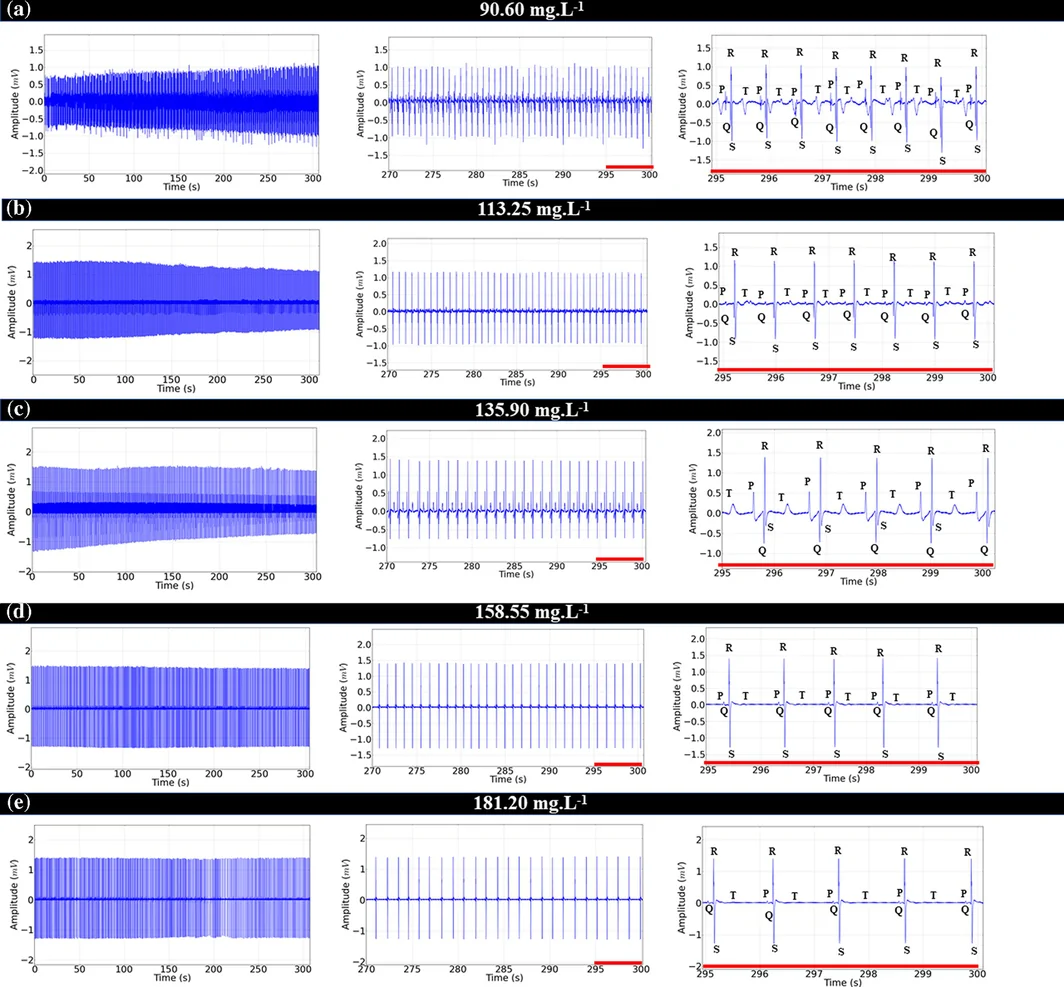
Cardiac activity in juvenile *Colossoma macropomum* during recovery following immersion baths with different concentrations of ROEO (left). Amplification of the recording during the last 30 s (270–300 s) for identification of cardiac deflections (centre) during the recovery period after immersion with the following concentrations of ROEO (5 s amplification) (right): 90.60 mg L^−1^ (a), 113.90 mg L^−1^ (b), 135.90 mg L^−1^ (c), 158.55 mg L^−1^ (d) and 181.20 mg L^−1^ (e).

For the group treated with 90.60 mg L^−1^ of ROEO, the heart rate recovered 99.48% compared to the control (Figure 6a). The recovery percentages for the other groups were as follows: 113 mg L^−1^ (96.16%) (Figure 6b), 135.90 mg L^−1^ (74.68%) (Figure 6c), 158.55 mg L^−1^ (68.54%) (Figure 6d) and 181.20 mg L^−1^ (66.49%) (Figure 7e).

During the recovery period, no arrhythmias were observed, and the reversal of ROEO effects on the heart was gradual and concentration‐dependent.

The amplitude of the QRS complex during recovery for the control group was 1.168 ± 0.27 mV, which was like the other groups (F [6, 56] = 0.375, *p* = 0.891) (Figure 8b).

**FIGURE 8:**
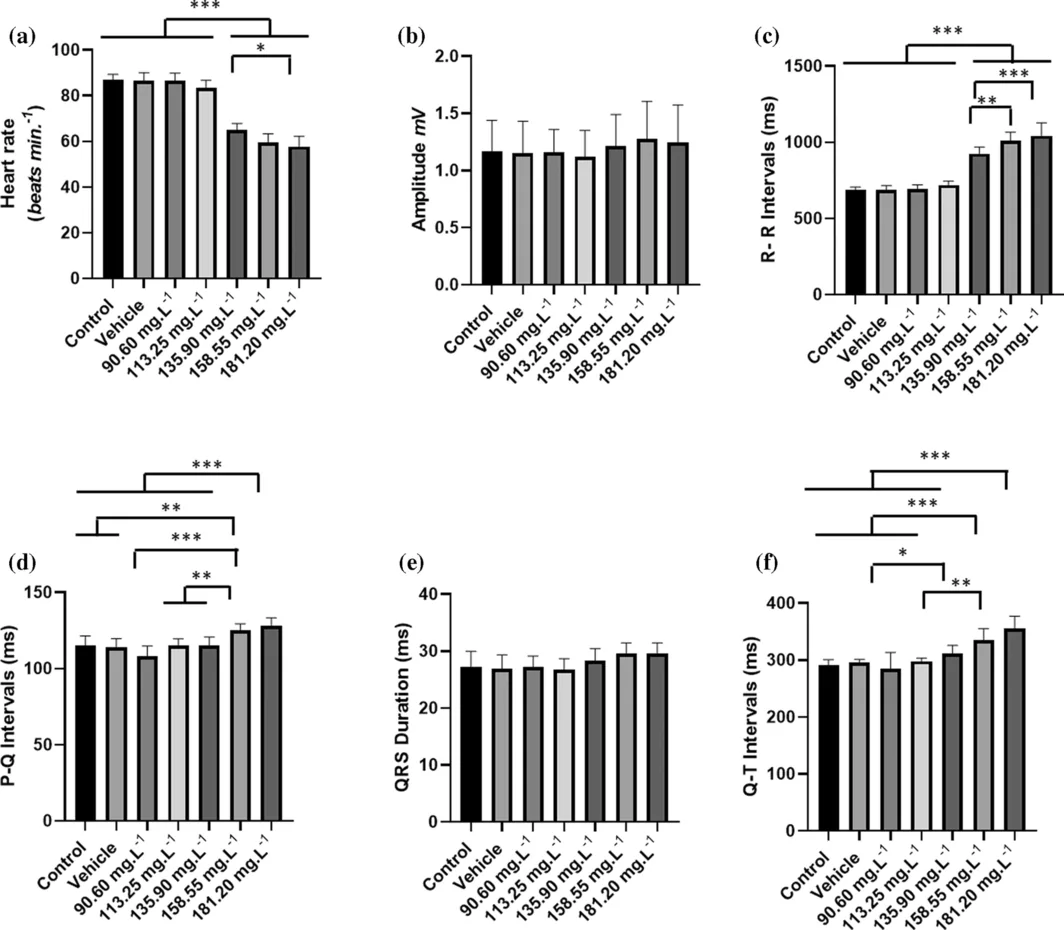
Mean values of cardiac parameters during recovery following exposure to different concentrations of ROEO in immersion baths at 90.60, 113.35, 135, 158.55, and 181.20 mg L^−1^. Mean values of heart rate (bpm) (a), amplitude of the QRS complex (mV) (b), RR interval (ms) (c), PQ intervals (ms) (d), duration of the QRS complex (ms) (e) and QT interval (ms) (f). (ANOVA followed by Tukey's test; *p < 0.05, **p < 0.01, ***p < 0.001; *n* = 9).

The average RR interval during recovery for the control group was 686.7 ± 19.33 ms, showing no statistical difference compared to the vehicle, 90.60 mg L^−1^ and 113 mg L^−1^ groups (*p* = 0.7572); however, it was shorter than the other groups. The groups treated with higher concentrations, 135.90 μL L^−1^ (926.1 ± 43.80 ms), were shorter than the 158.55 mg L^−1^ (1009 ± 57.90 ms) and 181.20 mg L^−1^ (1043 ± 85.05 ms) groups. The 158.55 mg L^−1^ and 181.20 mg L^−1^ groups were similar (*p* = 0.5171) (Figure 8c).

The PQ interval during recovery for the control group was 115.1 ± 6.27 ms, which was like the vehicle, 90.60 mg L^−1^, 113 mg L^−1^ and 135 mg L^−1^ groups (*p* = 0.1260). The group treated with 158.55 mg L^−1^ (125.3 ± 4.062 ms) was like the group treated with 181.20 mg L^−1^ (*p* = 0.9543) but was longer than the other groups (Figure 8d).

The duration of the QRS complex during recovery for the control group averaged (27.22 ± 2.72 ms) and was like the other groups (*F* [6, 56] = 2.88; *p* = 0.0161) (Figure 8e).

During recovery, the QT interval for the control was 290.3 ± 10.51 ms, which was like the vehicle, 90.60 mg L^−1^, 113.25 mg L^−1^ and 135.90 mg L^−1^ groups (*p* = 0.1525). The groups treated with 135.90 mg L^−1^ (311.8 ± 14.06 ms) were like the 158.55 mg L^−1^ group (*p* = 0.1142). The group treated with 181.20 mg L^−1^ (354.9 ± 22.27 ms) was like the group treated with 158.55 mg L^−1^ (*p* = 0.1894) (Figure 8f).

During the treatment with ROEO at concentrations of 90.60 mg L^−1^, 113.25 mg L^−1^, 135.90 mg L^−1^, 158.55 mg L^−1^ and 181.20 mg L^−1^, the frequency of opercular movement decreased with higher concentrations (Figure 9a–g). The control group exhibited a mean of 98.67 ± 6.92 movements per minute (mpm), which was similar to the vehicle group (*p* = 0.9282) but higher than the other treatment groups. The group treated with 90.60 mg L^−1^ showed a reduction in opercular beats of 22.52%. The group treated with 113.25 mg L^−1^ exhibited a decrease of 21.17%, while the group treated with 135.90 mg L^−1^ showed a reduction of 31.08%. The group treated with 158.55 mg L^−1^ reduced opercular activity by 32.43%, and the group treated with 181.20 mg L^−1^ demonstrated a decrease of 37.95%.

**FIGURE 9:**
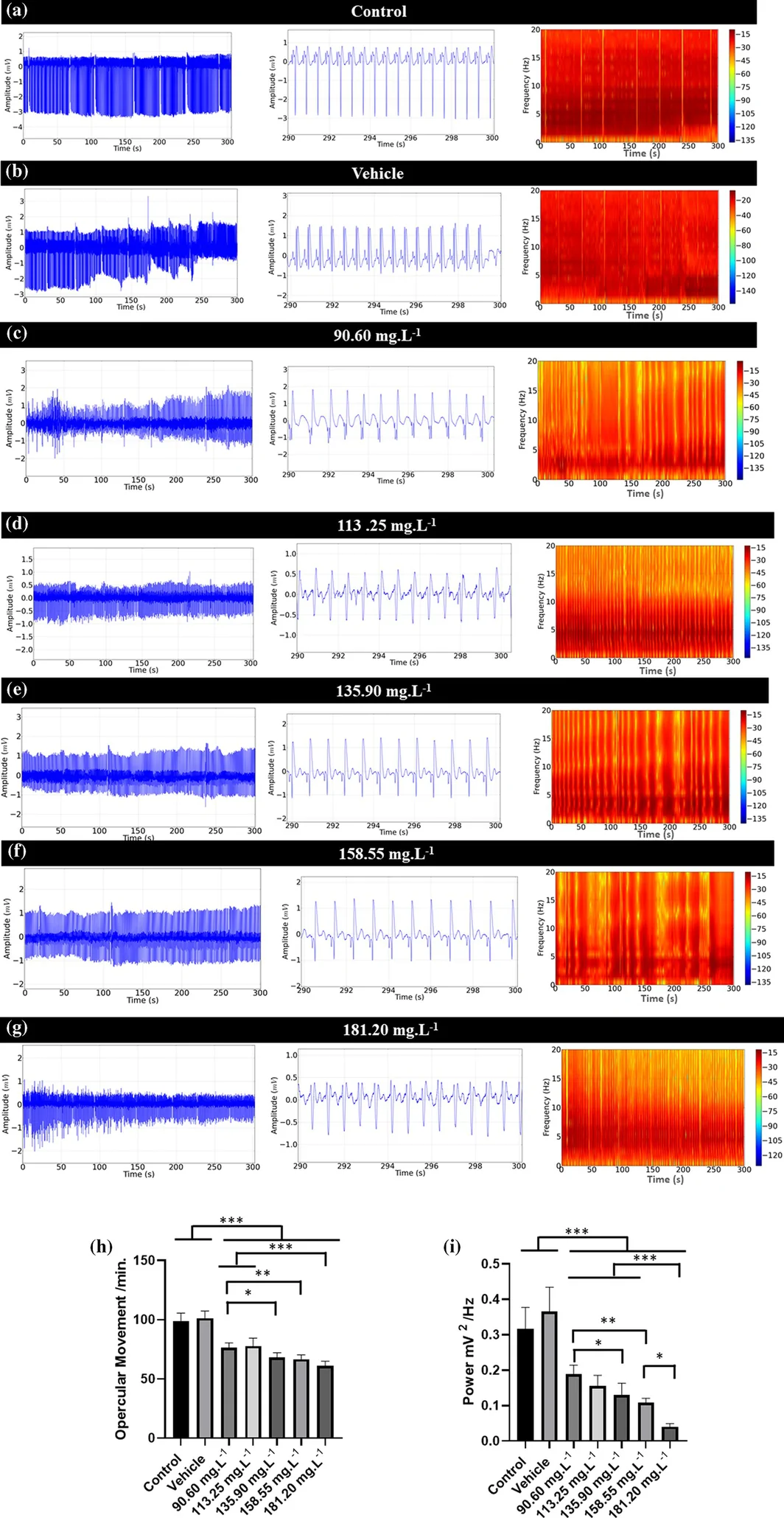
Record of opercular activity in juveniles of *Colossoma macropomum* during immersion in different concentrations of ROEO (left). Amplification of the record over the last 10 s (290–300 s) (centre); spectrogram of energy distribution during immersion at different concentrations of ROEO (right). Observations were made for the following groups: Control group (a); Vehicle group (b); Group treated with 90.60 mg L^−1^ (c); Group treated with 113.25 mg L^−1^ (d); Group treated with 135.90 mg L^−1^ (e); Group treated with 158.55 mg L^−1^ (f) and Group treated with 181.20 mg L^−1^ (g). It presents the mean values of opercular movement per minute (mpm) (h) and the mean values of opercular movement power (mV^2^/Hz) (i). (ANOVA followed by Tukey's test; *p < 0.05, **p < 0.01, ***p < 0.001; *n* = 9).

The group treated with 90.60 mg L^−1^ (76.44 ± 3.97 mpm) was like the group treated with 113.25 mg L^−1^ (*p* = 0.9980), but higher than the other treatment groups. The group treated with 135.90 mg L^−1^ (68.00 ± 4.123 mpm) was like the groups treated with 158.55 mg L^−1^ and 181.20 mg L^−1^ (*p* = 0.0995) (Figure 9h).

The decrease in power in the opercular movement records was observed during the induction of anaesthesia. The control group showed a mean power of 0.3165 ± 0.0609 mV^2^/Hz, which was similar to the vehicle group (*p* = 0.134) but greater than that of the other treatment groups. The group treated with 90.60 mg L^−1^ showed a mean of 0.189 ± 0.025 mV^2^/Hz, which was like the group treated with 113.25 mg L^−1^ (*p* = 0.5722). The group treated with 113.25 mg L^−1^ (0.155 ± 0.0301 mV^2^/Hz) was like the groups treated with 135.90 mg L^−1^ and 158.55 mg L^−1^ (*p* = 0.1882). The group treated with 181.20 mg L^−1^ (0.04063 ± 0.0087 mV^2^/Hz) was lower than the other groups (Figure 9i).

During the recovery period from anaesthesia, opercular movement showed recovery (Figure 10a–e). The control group had a mean of 98.67 ± 6.92 mpm, which was like the vehicle group, the group treated with 90.60 mg L^−1^, the group treated with 113.25 mg L^−1^ and the group treated with 135.90 mg L^−1^ (*p* = 0.1338), but higher than the other treated groups. The group treated with 158.55 mg L^−1^ (81.67 ± 5.52 mpm) was like the group treated with 181.20 mg L^−1^ (*p* = 0.2669) (Figure 10f).

**FIGURE 10:**
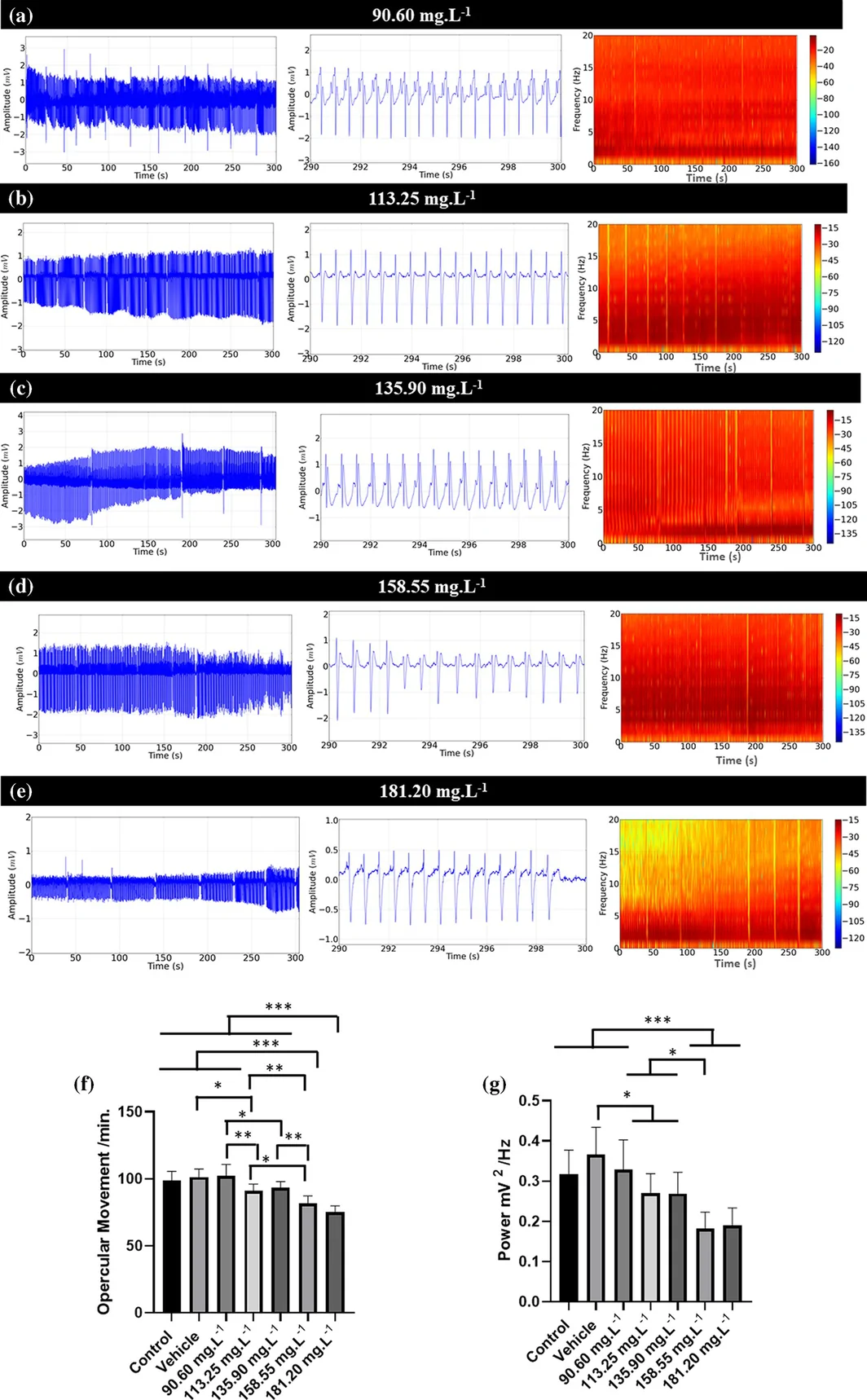
Opercular movement recording in juvenile *Colossoma macropomum* during recovery after immersion baths with different concentrations of ROEO (left). Amplification of the recording in the last 10 s (290–300 s) to identify the movements (centre), and a spectrogram indicating the energy level during the animals' recovery (right). Data were obtained after immersion baths with the following concentrations: 90.60 mg L^−1^ (a), 113.25 mg L^−1^ (b), 135 mg L^−1^ (c), 158 mg L^−1^ (d) and 181.20 mg L^−1^ (e). Mean values of opercular movements per minute (f); mean power values of opercular movement (mV^2^/Hz) (g). (ANOVA followed by Tukey's test; *p < 0.05, **p < 0.01, ***p < 0.001; *n* = 9).

An increase in power in the opercular movement recordings was observed during recovery from anaesthesia. The control group had a mean power of 0.3165 ± 0.0609 mV^2^/Hz, which was like the recovery groups of the vehicle, treated with 90.60 mg L^−1^, 113 mg L^−1^ and 135.90 mg L^−1^ (*p* = 0.5085). The group treated with 158.55 mg L^−1^ was like the group treated with 181.20 mg L^−1^ (*p* = 0.0549). The group treated with 181.20 mg L^−1^ was like the groups treated with 135.90 mg L^−1^ and 158.55 mg L^−1^ (*p* = 0.0603) (Figure 10g).

## 4 DISCUSSION

The results of this study demonstrate that *Rosmarinus officinalis* essential oil (ROEO) exerts a concentration‐dependent anaesthetic effect on *Colossoma macropomum* (tambaqui). The group treated with 182.20 mg L^−1^ of essential oil showed a significantly faster loss of postural reflex compared to groups with lower concentrations, such as 90.60 mg L^−1^, indicating a faster anaesthetic action at higher concentrations. These findings align with the study by Bianchini et al. (2017), which evaluated the anaesthetic effects of essential oil compounds like carvacrol and thymol and observed a clear relationship between concentration and anaesthetic response, with significantly shorter induction times as concentrations increased across all induction stages.

Uehara et al. (2019), in a comparative study of synthetic and natural anaesthetic agents, highlighted that clove oil exhibited effective anaesthetic action across all tested concentrations, with dose‐dependent effects on both anaesthetic induction and recovery time. Similarly, the lower concentrations of *R. officinalis* essential oil evaluated in this experiment, such as 90.60 mg L^−1^ and 135.90 mg L^−1^, required a longer latency time for anaesthetic action than the other concentrations. Meanwhile, postural reflex recovery time was longer in groups treated with higher doses, demonstrating the dose‐dependent and gradual reversibility of the anaesthetic effect—a characteristic often described in the literature for natural anaesthetics (Hoseini et al., 2019).

These observed effects may be attributed to the composition of ROEO, particularly its main component, 1,8‐cineole, whose action on the central nervous system is well‐documented in the literature. High‐performance gas chromatography analysis revealed that 1,8‐cineole comprises 47.5% of the oil composition, followed by camphor (19.3%) and α‐pinene (12.2%). These terpenoid compounds are known for their biological properties, including antioxidant and anti‐inflammatory activities and synergistic effects in various applications. The anaesthetic effect of *R. officinalis* is closely linked to 1,8‐cineole, which modulates GABA‐A receptors in the central nervous system and blocks voltage‐dependent sodium channels in peripheral nerves, resulting in reduced neuronal excitability (Dougnon & Ito, 2020). Studies such as Khumpirapang et al. (2018) have demonstrated the potent depressant effect of 1,8‐cineole on the central nervous system, explaining the rapid anaesthetic induction observed at higher ROEO concentrations.

Additionally, 1,8‐cineole significantly impacts the cardiovascular system, inducing bradycardia and modulating cardiac electrical activity. In this study, prolonged R‐R and QT electrocardiographic intervals were observed, like effects reported in fish treated with other essential oils containing 1,8‐cineole or camphor (Mazandarani & Hoseini, 2017). The reduction in QRS complex amplitude at higher doses (181.20 mg L^−1^) may be associated with ventricular depression caused by *R. officinalis*, indicating a direct effect on heart contractility. Studies with essential oils, such as eucalyptus oil, have also reported reduced QRS complex amplitude, suggesting that these compounds may modulate cardiac electrical activity, affecting myocardial cell function (Mirghaed et al., 2018).

The extension of the R‐R interval observed at higher ROEO doses suggests modulation of the interval between heartbeats, consistent with induced bradycardia. These findings indicate that ROEO affects the cardiac cycle in a concentration‐dependent manner, similar to findings in studies with clove oil and lemongrass (Bianchini et al., 2017). Furthermore, the increase in the P‐Q interval and prolonged QRS complex duration at higher ROEO doses indicate delayed atrioventricular conduction, possibly related to the action of 1,8‐cineole on calcium channels and the cardiac conduction system (Aktop et al., 2019). This effect is observed in other natural anaesthetics and should be considered a critical parameter to determine safe doses, avoiding cardiac blocks or prolonged alterations in fish cardiac electrical conduction.

Prolongation of the QT interval at higher ROEO doses suggests increased ventricular repolarization time, which may indicate a risk of arrhythmias if used at inappropriate concentrations. This finding is relevant because anaesthetics that prolong the QT interval are often associated with a higher risk of arrhythmias (Schroeder et al., 2021). However, the reversibility of the effect after recovery, without arrhythmias, demonstrates that ROEO is safe within the tested dose range.

The electrocardiographic data show that, for all concentrations of *R. officinalis*, there was a significant dose‐dependent reduction in heart rate, but sinus rhythm was maintained, indicating that *R. officinalis* did not compromise the normal electrical function of the heart. This suggests that ROEO can be considered a safe anaesthetic for use in fish, with a cardioprotective effect and no arrhythmias. These results support the findings of Krasteva et al. (2021), who evaluated *R. officinalis* oil in *Cyprinus carpio* L. and found that, in addition to dose‐dependent anaesthetic induction, all oil concentrations were safe for fish transport for up to 4 h without causing harm to the animals.

The complete reversibility of the electrocardiographic changes observed during recovery with ROEO reinforces its safety profile for aquaculture, with sinus rhythm being progressively restored. This is especially advantageous, as natural anaesthetics, like ROEO, often exhibit slower recovery due to their prolonged bioavailability (Can & Sümer, 2019; Percie du Sert et al., 2020). The near‐total recovery of heart rate at lower concentrations also suggests that moderate ROEO doses can induce effective anaesthesia without compromising long‐term cardiovascular function. In contrast, MS‐222 causes an abnormal respiratory response in fish, with rapid and irregular gill movements, indicating respiratory depression and stress. This abrupt behaviour contrasts with essential oils, which tend to promote a smoother, more controlled anaesthetic induction, reducing stress during the process and highlighting the safety of essential oils as natural anaesthetics in aquaculture.

This study demonstrated that *Rosmarinus officinalis* essential oil exerts a dose‐dependent anaesthetic effect on *Colossoma macropomum*. The findings also indicated that the main component of ROEO, 1,8‐cineole, plays a key role in modulating the central nervous system and regulating cardiac activity. Electrocardiographic data revealed changes such as bradycardia and prolongation of the R‐R and QT intervals, effects that were gradually reversed during recovery, reinforcing the safety of *R. officinalis* as a natural anaesthetic. *R. officinalis* provided a gentler anaesthetic induction with low impacts on the respiratory function of the fish, providing a more controlled anaesthetic transition. These findings indicate that *R. officinalis*, in addition to being effective, is a safer and more sustainable alternative for fish management in aquaculture, offering an option that minimizes the side effects associated with synthetic anaesthetics.

Given the demonstrated efficacy and safety of *R. officinalis*, future studies may focus on assessing its impact on other fish species and examining its use under long‐term conditions, such as for prolonged fish transport and handling. Research on the elimination of *R. officinalis* compounds and potential bioaccumulation may also provide important insights to ensure the safety of continued use. Furthermore, further studies on optimizing *R. officinalis* concentrations may help establish clear guidelines for its routine use in aquaculture, consolidating its potential as a natural anaesthetic. In this study, we observed the reversibility of its effects in concentration‐dependent anaesthesia; therefore, we can indicate concentrations of 90.60–135.90 mg/L^−1^ for superficial anaesthesia and 158.55–181.20 mg L^−1^ for deep anaesthesia in a short‐duration immersion bath.

## AUTHOR CONTRIBUTIONS

Lorena Cristina Nunes de Almeida and Moisés Hamoy conceived and designed the experiments. Daniella Bastos de Araújo, Axell Lins and Moisés Hamoy performed the experiments. Axell Lins, Maria Klara Otake Hamoy, Marcelo Victor dos Santos Brito, Luana Vasconcelos de Souza, Sarah Farias Câmara, Luciana Eiro Quirino and Nilton Akio Muto contributed to writing the original draft and editing. Moisés Hamoy provided financial support and administrative support. All authors have read and agreed to the published version of the manuscript.

## FUNDING INFORMATION

This work was supported by the Federal University of Pará (UFPA), through the Laboratory of Pharmacology and Toxicology of Natural Products, and by internal research grants from the Graduate Program in Biological Sciences (ICB‐UFPA). No external funding agencies influenced the study design, data collection or interpretation.

## CONFLICT OF INTEREST STATEMENT

The authors declare no conflicts of interest.

## Data Availability

The datasets generated and analysed during the current study (raw ECG traces, opercular activity records and chromatographic profiles) are available from the corresponding author upon reasonable request. Processed data and scripts for signal analysis (Python 2.7, NumPy, SciPy and Matplotlib) are stored in the institutional repository of the Federal University of Pará and can be provided for review or replication purposes.
